# Supplementary material for: Patterns of Intron Gain and Loss in Fungi
Source: PLoS Biol. 2004 Nov 30;2(12):e422. doi: 10.1371/journal.pbio.0020422 (PMC532390; doi:10.1371/journal.pbio.0020422)
Supplement: Table S1 — Also available at http://genes.mit.edu/NielsenEtAl/. (4.3 MB ZIP). [file pbio.0020422.st001.zip › NielsenEtAl/html/699.html]

AN0314.1.NCU07082.1.MG04681.1.FG01976.1


```
 CLUSTAL W (1.82) Multiple Sequence Alignments - Introns Inserted


Sequence 1: MG04681.1	1150 aa
Sequence 2: FG01976.1	1033 aa
Sequence 3: AN0314.1	956 aa
Sequence 4: NCU07082.1	915 aa
Alignment Length: 1290 aa
Number Identitical Residues: 269 aa
Alignment Score (without introns) 15131


MG04681.1 	--~-MYTARKSHSPLLCTSIAAD~PKAAE---------------NKPINSIS----GLFS
NCU07082.1	--~---MSPG--------KLKKP~LLALN---------------RIIGHSSA--------
FG01976.1 	MP2NYSAAPDSSFLILLNQLQAT0PRSSRPVPDSESPKRELNNRKQPARSSSTASRGYFN
AN0314.1  	--~---MSIKRALSKIKPKLEEN~LPSPS---------------SSRQGSSS--------
          	       :          .:      :                      * :        

MG04681.1 	QLLVSSKAG------SFKVVHNHSLTELITGPIR~KATSRTSTPVQSP------------
NCU07082.1	--------------------KNHMSKPKIG----~--------DIDQL------------
FG01976.1 	SGNITESQNQDRHIQDFKVWLTHRATPSIIIAYC1LATTTKNSPTSSPSTPTSSTTPRFP
AN0314.1  	--------------------RTSSPRRNILSSFL~RDRDSASSSDDIS------------
          	                     .      *  .         .:  .              

MG04681.1 	-------------------------VACHTTNSQTS-----------------------P
NCU07082.1	----------------------------DDERAKKN------------------------
FG01976.1 	SPLITSFTAAPATASRPLSTISIDSVLSEPTTTLTSSSFYTTQMASPDTTRRPASVNMSP
AN0314.1  	----------------------------DDASGAGS------------------------
          	                            .      .                        

MG04681.1 	RSKSRGHR------SSDDDSYRSDSLERKARKEAD----KLERRLQEQELIDARRRSED-
NCU07082.1	IQKIQEKR----------AKEYQLEEEKKLMIQK--------RSEEEKRWHEADG-----
FG01976.1 	APISVDTRPSAAILSSAFSSPRSVSPSRTIRETMNDSGVELPHRESARSSMDGTTATTNA
AN0314.1  	VSKNQQKR----------LARRQRKSEQRSRMSED----IGGSSESERRHKEEIDQAIRE
          	       *              . . .:      .          . :   :    :   

MG04681.1 	--LKAREHLDEATRARFGDDVVFR-ELTPIAQLVTLPVGSEITFRARIHHQRDISKALDF
NCU07082.1	--PEIARRY--GTQ---EEEALAPAKSLQVLVNDYQNIGKTVSFRARVHHVRALSSKLAF
FG01976.1 	IVPSSRTSLDSGRTSMSSDWPVHPGEINTIAQLDTLPFGTEVTFRARIETQRPISKVLDF
AN0314.1  	ETPEMRARY--GELPLLQSSTRKREKRLDFDTISADMVGQQVFFQARLHIIRRMSAKLVF
          	   .       .  .   .      :   .       .*  : *:**:.  * :*  * *

MG04681.1 	LLLRDQTHSIQGVLQ-----GPPEMVKWAQRLHPESIVQITGHLQCPREPVRSATHQGLE
NCU07082.1	VVFRDQIETVQGVLSVRDGVVSENFVRWAEHITAESFVHVEGKLQQPPEEIKGCTIHNVE
FG01976.1 	LLLRDQTHSVQGVLARD--AGNADFISWVRKINSESLVQITGTLKQPPEPIRSATHSGVE
AN0314.1  	LVFRQQLSTFQGVLHEEPGAKSIAMIQWAEHLKTGCIVQVRGTIKAPDVPVLGCTVHDVE
          	:::*:*  :.****    .     :: *..:: . .:*:: * :: *   : ..*  .:*

MG04681.1 	VLIDTIHLVAPAKN-LSFNNYQPPE---------------SLHPRLTNRVVDLRHPANQA
NCU07082.1	VLIDTMHLVVPIDEHLPVDVHSIDHVEVDSETN-QLESLASTRVRVSNRIAFLRTPTAQS
FG01976.1 	VAVDSVHLVNLAQN-LPFSNYKPPE---------------TLRNRMNARILDLRHPSNQA
AN0314.1  	VDIDEVHLVVRREDPVPFSVYEAEIRTAEEDLVEGRRSHISDRTRLTNRLLDLRTPTSQS
          	* :* :***   .: :... :.      ...  .   :  : : *:. *:  ** *: *:

MG04681.1 	LFRVRAAVTRTFRWTLDQQDFVEIQTPKIMAGATESGAEVFKVNYFGRSAYLAQSPQLSK
NCU07082.1	IFRINAGICSIFRRYLEDKGFMEIHTPKLQPAATESGAEVFKVNYFGRTAFLAQSPQLAK
FG01976.1 	LFRVRAMVTRVFRNTLEEHGFVEINTPKLQPAATESGAAVFPVNYFGRRAFLAQSPQLAK
AN0314.1  	IFRIQSAVCNLFRSALDEQRFIEIHTPKLQGAATESGASVFDVNYFGRDAFLAQSPQLAK
          	:**:.: :   **  *::: *:**:***:  .****** ** ****** *:*******:*

MG04681.1 	QMAISADFKRVYE0VGPVFRAENSNTHRHLTEYTGLDIEMCIDQSHHEIIRMVDRVLKNI
NCU07082.1	QMSISADFGRVFE~IGPVFRAEDSNTHRHLTEYTGMDLEMAIEQDYHEAMHIIDGLMKEI
FG01976.1 	QEAISADFGRVFE~VGPVFRAENSNTHRHLTEYTGLDLEMAIDTDYHEVISFIDIFLKEV
AN0314.1  	QMAIAADFGRVYE~IGAVFRAENSNTHRHLTEYTGLDIEMSIEEHYHEMLEVLDAVIKNI
          	* :*:*** **:* :*.*****:************:*:**.*:  :** : .:* .:*::

MG04681.1 	FAAVH--QMEELKIIRERWPSEDLVWLEETPILTFPEALQMLR------EDGNEDIEEDD
NCU07082.1	FKGIYGKYRKEIEIIKTRFPHEDLVWLPETPIIPFKDAIKLLNDSGWTDDHGRPALETED
FG01976.1 	FRTVY--ASRELEVIRKRWPSKEFKWLEETLVLPFSEGLQMLR------DDGRE-VEVED
AN0314.1  	LKGIYTRYRREIEIIKHQFPSEDLVWLDETPIIRFSDGIKMLNESGWRDDEGNPLAEDKD
          	:  ::    .*:::*: ::* ::: ** ** :: * :.:::*..:.  .:.*.   * .*

MG04681.1 	LSTRDEYRLGELVKEKYKTDYFILDKFPTIARPFYTHKDDSDPKWTNSFDIFVRGQEICT
NCU07082.1	LSTRAEIQLGQVIKEKYKTDYYIIDKFPSSVRPFYTHLDPEDDRFTNSFDIFLRGQEITT
FG01976.1 	LSTPDEMRLGELVRQKYDTDYYVLDKFPANARPFYTAKDPEDPTWTRSFDIFIRGQEICS
AN0314.1  	LGTRDEIRLGELVKEKYKTDYYVLDKFPVDARPFYAMPDPDDPRFTNSFDMFIRGQEIVS
          	*.*  * :**:::::**.***:::****  .****:  * .*  :*.***:*:***** :

MG04681.1 	GGQRIHKYDELRQSMTDSGISGDGMEEYLAAFESGAPPHGGAGLGLERIITFMLQLGDVR
NCU07082.1	GGQRIHRPHLLEERMKKAGIEPLGMQEYLQGFEYGVLPHAGCGIGLERIVFLLLNLGDIR
FG01976.1 	GGQRIHNVDELRANMAEAGMAEDGMEDYLTAFELGAPPHAGAGLGLERIVAWMLELGDVR
AN0314.1  	GGQRIHDPHMLEENMRRVGINPDTMEEYMEGFRWGAPPHAGAGIGLERMLMLILKLGNIR
          	******  . *.  *   *:    *::*: .*. *. **.*.*:****::  :*:**::*

MG04681.1 	NAAPFHRDPKSLPSRPPGL---PHPEASTRRRKSATAPASQNNKESLHGTESSDMTPKSS
NCU07082.1	NASLIPRDPKSLPEHKELVGKLPHPEADTIKYAYDYDHG--------------------A
FG01976.1 	YASLFHRDPKSLPTKAPGL---PHPEADTTKPHHADSP----------------------
AN0314.1  	LASMFHRDPKSFPAKPPAL-QLRHPESSTIEPPWVKERRGT----------------VAQ
          	 *: : *****:* :   : .  ***:.* .        .                  : 

MG04681.1 	AETEYPPLEKLIANYGDATNTSWLDDRFQIWRHSPTGAAVGYVRQAKDLVIMTGDPLCDP
NCU07082.1	TGLELPPVEKLIVNYGDATNTSWLDERYHVWRHEQTGAAIGYAED-NGYALVMGNPLCDP
FG01976.1 	-----PPLEKLIANYGDATNTSWLDERFQIWRH-ETGAAIGWVQREK-FAMITGDPLCDR
AN0314.1  	SESELQPIEHLIANYGDATSTSWGDERFKIWRDMATGAAVSYVVSSNNYVVIPGNPLCDA
          	:  .  *:*:**.******.*** *:*:::**.  ****:.:.   :. .:: *:**** 

MG04681.1 	TQYAEIIGAYLDFVRNTLKCKPVWLLVSEEVQRILANEPHNWRTLTCNEEQRIPADDQAR
NCU07082.1	RQYQLVIRAFLQDMRHTKDLRPLWLLVGSEVEEILGSKLG-WRSLSCVAEERVS-----V
FG01976.1 	NQYPQVIKAFIHHITVDLHLTPFWMLVSFEVQKILASELR-WRSLTCTEEQRVDADKHNS
AN0314.1  	RQYGRVISQFLQWLRRETKYRPIWLLCSPEVETVLGEKLG-WRSLSCIAEEVVEPARNQA
          	 **  :*  ::. :    .  *.*:* . **: :*..:   **:*:*  *: : .     

MG04681.1 	RGLSRPGAQRSARRVQREGIRLHEVRPE--KDENFKQRANKAIDEWYRAREGKKQVHLTE
NCU07082.1	DSAKKVAKK--ERQAEEAGVSIHEVPIGEPVPEHIREKCDKRIQDWKDNRKGKKQVHITE
FG01976.1 	AQIEGLAAK--SRRVEREGVKIHEVK----ADEDFIARANPAIEEWKNNRKGK-QVHLTE
AN0314.1  	AMDGELARK--IRRTENEGIKIVTINQGEMVSESVREAIDKRIEDWKANRKG-TQVHLSD
          	      . :   *:.:. *: :  :   .   * .    :  *::*   *:* .***:::

MG04681.1 	VRPWIDAEHRRYFAAE-RDGRVLSLVVLHRLAPRYGWQVKWALDMPSAPNGTIEALVDLA
NCU07082.1	VRPWVDMEHRRYLWAEDKNGEIVAMCVLARLSPANGYQIKFALDFPGSPNGTIEALISAA
FG01976.1 	VRPWIDQEHRRYFAAE-KDGKVMSMVVLAKLAPRHGWQVKWALDFPGAVNGAIEVLVSFA
AN0314.1  	IHPWRDHEHRWYFYAVDKEGTICAFVTLVLISPTYGMQVKYTFDFPGSPNGVIESLITHA
          	::** * *** *: * .::* : :: .*  ::*  * *:*:::*:*.: **.** *:  *

MG04681.1 	LMSISGS---PATFGADVSGKLVPVAHIGGAKTKILTKAYETFVKGTGISRKKDFRGKFG
NCU07082.1	IQMLAKAGVRNVTFGAGALPEMVTGGHLNGMRAKMLSHTYKTICQQLKLVNKSEFREKFG
FG01976.1 	LSNVTG----KVTFGAGVSEKLTAGEHVGGLRAKFLSATYRSIIDSLGLRRKASFRSKFG
AN0314.1  	IQTAGRSGVKKLTFGAGATNTLTPGHNMHGAKMKMLQHTYDALAKQFHLVRKSEFRAKLG
          	:     :.    ****..   :..  :: * : *:*  :* :: .   : .* .** *:*

MG04681.1 	IVGEAVFICYPRGAMGMGEIREIYR2VNLTTQKRLAASVLDCGKNKIWLDPNEVSEISNA
NCU07082.1	ATNDLVYICYPFMGLGVSGVRTLIK~FFEDEM----------------------------
FG01976.1 	ALGEEIYICYPKKGVGLRDLQNVIK~FFTD------------------------------
AN0314.1  	AEDEPLYIAYPPHGLGQKGIRAILH~FFED------------------------------
          	  .: ::*.**  .:*   :: : : .                                 

MG04681.1 	NSRQTIRKLVQDGLIIKKPVTMHSRARARELNLARRIGRHRGFGKRKGTADARMPT2QVV
NCU07082.1	--------------------------------------------------------~---
FG01976.1 	--------------------------------------------------------~---
AN0314.1  	--------------------------------------------------------~---
          	                                                            

MG04681.1 	WMRRQRVLRRLLVKYRASGKIDKHLYHELYHSAKGNTFKHKRALVEH0IHRAKAEKQRER
NCU07082.1	-----------------------------------------------~------------
FG01976.1 	-----------------------------------------------~------------
AN0314.1  	-----------------------------------------------~------------
          	                                                            

MG04681.1 	LLKDEMDAKRAKTKAARERKLERQAAKRNALAGEAEE
NCU07082.1	-------------------------------------
FG01976.1 	-------------------------------------
AN0314.1  	-------------------------------------
          	
```
